# A Preclinical Model of Computerized Cognitive Training: Touchscreen Cognitive Testing Enhances Cognition and Hippocampal Cellular Plasticity in Wildtype and Alzheimer’s Disease Mice

**DOI:** 10.3389/fnbeh.2021.766745

**Published:** 2021-12-06

**Authors:** Amy Shepherd, Tracy Zhang, Lucas B. Hoffmann, Ariel M. Zeleznikow-Johnston, Leonid Churilov, Anthony J. Hannan, Emma L. Burrows

**Affiliations:** ^1^Florey Institute of Neuroscience and Mental Health, Melbourne Brain Centre, University of Melbourne, Parkville, VIC, Australia; ^2^Melbourne Medical School, The University of Melbourne, Parkville, VIC, Australia; ^3^Department of Anatomy and Neuroscience, The University of Melbourne, Parkville, VIC, Australia

**Keywords:** touchscreen, mice, cognitive enhancer, Alzheimer’s disease, APP/PS1

## Abstract

With the growing popularity of touchscreen cognitive testing in rodents, it is imperative to understand the fundamental effects exposure to this paradigm can have on the animals involved. In this study, we set out to assess hippocampal-dependant learning in the APP/PS1 mouse model of Alzheimer’s disease (AD) on two highly translatable touchscreen tasks – the Paired Associate Learning (PAL) task and the Trial Unique Non-Matching to Location (TUNL) task. Both of these tests are based on human tasks from the Cambridge Neuropsychological Test Automated Battery (CANTAB) and are sensitive to deficits in both mild cognitive impairment (MCI) and AD. Mice were assessed for deficits in PAL at 9–12 months of age, then on TUNL at 8–11 and 13–16 months. No cognitive deficits were evident in APP/PS1 mice at any age, contrary to previous reports using maze-based learning and memory tasks. We hypothesized that daily and long-term touchscreen training may have inadvertently acted as a cognitive enhancer. When touchscreen-tested mice were assessed on the Morris water maze, they showed improved task acquisition compared to naïve APP/PS1 mice and wild-type (WT) littermate controls. In addition, we show that touchscreen-trained WT and APP/PS1 mice show increased cell proliferation and immature neuron numbers in the dentate gyrus compared to behaviorally naïve WT and APP/PS1 mice. This result indicates that the touchscreen testing paradigm could improve cognitive performance, and/or mask an impairment, in experimental mouse models. This touchscreen-induced cognitive enhancement may involve increased neurogenesis, and possibly other forms of cellular plasticity. This is the first study to show increased numbers of proliferating cells and immature neurons in the hippocampus following touchscreen testing, and that touchscreen training can improve cognitive performance in maze-based spatial navigation tasks. This potential for touchscreen testing to induce cognitive enhancement, or other phenotypic shifts, in preclinical models should be considered in study design. Furthermore, touchscreen-mediated cognitive enhancement could have therapeutic implications for cognitive disorders.

## Introduction

Touchscreen-based cognitive testing methods have been developed for rodents in an effort to substantially improve translation between preclinical studies and clinical trials (Palmer et al., 2021). The technique provides a more translational approach to rodent cognitive testing, mimicking the stimuli (images in different locations on the screen) and reaction (touch) of those employed in human assessment methods, such as the Cambridge Neuropsychological Test Automated Battery (CANTAB). Touchscreen testing has been widely adopted as a method to assess cognitive decline in preclinical animal models of Alzheimer’s disease (Romberg et al., 2011, 2013; Piiponniemi et al., 2017; Shepherd et al., 2019, 2021; Van den Broeck et al., 2019; Saifullah et al., 2020). Specifically, this paradigm has great utility for non-invasive early detection of impairments in these animal models, enabling elucidation of mechanism of disease progression and screening of novel therapeutics (reviewed in Shepherd et al., 2016; Palmer et al., 2021).

Tasks such as paired-associate learning (PAL) and trial-unique-non-matching to location (TUNL) have been used to track hippocampal-dependent memory loss in a number of AD animal models (Saifullah et al., 2020). Additionally, reversal learning tasks have been sensitive to cognitive flexibility impairments in AD mouse models as they age (Piiponniemi et al., 2017; Shepherd et al., 2019; Van den Broeck et al., 2019) and attention and executive control have also been scrutinized and shown to be sensitive to donepezil (Romberg et al., 2011), a drug commonly used clinically for AD. Recently, Van den Broeck et al. (2021) showed that touchscreen tests were more sensitive in picking up memory impairments in an AD model compared to traditional maze-based tests. Although this is promising for early detection of impairments, and thus insight into early disease mechanisms, not all studies have shown early detection of impairments, with one reporting no differences in aged mice expressing a mutation in Tau (Kent et al., 2018). While touchscreen testing has been widely adopted since its development (Bussey et al., 2012), with over 300 research groups worldwide now using this paradigm (Dumont et al., 2020), traditional maze-based methods still dominate AD preclinical research. In order to advance our understanding of cognitive function in AD mouse models, it is critical that we understand how outcomes from both traditional and touchscreen methods of assessment compare to each other. Importantly, a number of unique and essential methodological components of touchscreen testing have documented effects on aging, memory, mood and brain health and thus exposure to the paradigm itself could have the potential to alter or shift behavioral phenotypes and neurobiology of the animals undergoing training.

Touchscreen training requires animals to respond to visual stimuli projected onto a touch-sensitive computer screen for a reward and relies on the natural exploration tendencies of rodents and daily training for animals to complete iterative steps that result in task acquisition. In order to motivate animals to complete many trials per daily session, caloric restriction (CR) in combination with a reward per trial is used. It has been well-documented that CR can rescue aging-induced cognitive deficits (reviewed in Van Cauwenberghe et al., 2016), increase synaptic plasticity markers (including adult hippocampal neurogenesis), and reduce markers of stress and inflammation (Lee et al., 2000, 2002). Given that touchscreen tasks require extended training to reach learning criteria, increasing the time under CR, this factor becomes a significant experimental manipulation. In addition, the daily increase in exercise and exposure to novelty during touchscreen training may be akin to environmental enrichment or running wheel paradigms, that expose animals to increased opportunities for cognitive and somatosensory stimulation as well as physical activity (reviewed in Nithianantharajah and Hannan, 2009). These paradigms have been extensively studied in the context of aging and AD, and both have established positive effects on cognition and brain plasticity (for review, see Shepherd et al., 2018). A number of studies have indicated that behavioral testing itself can have positive effects on the rodent brain compared to naïve controls (Billings et al., 2007; Martinez-Coria et al., 2015) and can boost performance on other cognitive tasks (Jiang et al., 2015; Martinez-Coria et al., 2015). With computerized cognitive training (CCT) gaining traction as a potential therapy to delay cognitive decline in older adults and in mild cognitive impairment (MCI) (Lampit et al., 2014; Hill et al., 2017), the potential for daily touchscreen training to mimic this intervention in preclinical rodent models warrants investigation.

In this study, we tested the hypothesis that extended touchscreen training could shift the cognitive phenotype of the commonly used APPswe/PS1ΔE9 (APP/PS1) mouse model of AD. APP/PS1 and WT mice were assessed in the hippocampal-dependent touchscreen tasks TUNL and PAL, undergoing a minimum of 3 months daily training. To investigate the effect of this extended training on cognitive function we compared touchscreen-trained WT and APP/PS1 animals to training-naïve mice in the hippocampal-dependent Morris water maze (MWM). Cell proliferation and number of immature neurons in the dentate gyrus were analyzed in all groups following cognitive assessment. With the increased adoption of touchscreen testing to assess cognitive decline in rodent models, this study aims to provide critical information regarding the potential impact of extended training on shifting phenotypes, transference to other tasks, and how touchscreen outcomes might be compared to other modes of assessment.

## Materials and Methods

### Animals

Male APPswe/PS1ΔE9 mice (Jax strain: 4462) and littermate controls on a 50:50 C57BL/6;C3H hybrid background were bred on site at the Florey Institute of Neuroscience and Mental Health. Groups of 2–4 mice were initially housed in individually ventilated cages (39 × 20 × 16 cm) with a small shelter on a 07:00-19:00 light cycle. At 8 months of age, groups were moved to open top cages (34 × 16 × 16 cm) and into a room with a reversed light cycle (19:00-07:00). Following transfer to their new environment, animals were weighed for 3 days to obtain a free-feeding weight (FFW), and then food restricted to achieve 85% FFW. Simultaneously, the mice began pre-training for behavioral testing. Female mice were not tested due to interference with male performance when they share touchscreen chambers. All procedures were approved by The Florey Institute of Neuroscience and Mental Health Animal Ethics Committee and were conducted in accordance with the Australian Code of Practice for the Care and Use of Animals for Scientific Purposes as described by the National Health and Medical Research Council of Australia.

### Apparatus

All touchscreen experiments were conducted in automated touchscreen-based operant systems (Campden Instruments Ltd, Loughborough, United Kingdom). Task deployment and automated event recordings were managed through the software Whisker Server and ABET II (Layfette Instruments, Layfette, IN, United States). Apparatus and task training methods have been published previously (Kim et al., 2015). MWM procedures were conducted in a 1.4 m wide and 0.5 m high pool with a 13 × 13 cm square platform, with 2D and 3D cues placed around the room as previously described (Highly salient cue set up, Rogers et al., 2017). The pool was filled with opaque water at 22 ± 2°C to approximately 30 cm, which submerged the platform by about 0.5–1 cm. Discrete-trial forced alternation, adapted from Deacon et al. (2003), was performed in a Y maze that was 7.5 cm wide, 13 cm high and had an arm length of 38 cm. The arms of the maze were at 120° angle. Proximal laminated paper cues (a black triangle and black cross) were placed on the two experimental arms, with no cue on the home arm. Topscan tracking software (Clever Sys, Restin, VA, United States) was used to track animals in both the Y-maze and MWM procedures.

### Study Design

**FIGURE 1:**
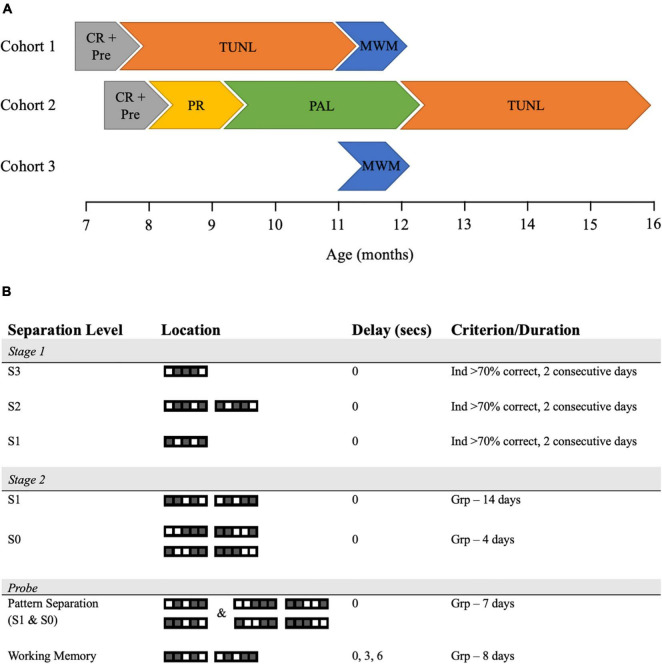
Cohort timings and TUNL task training and probe parameters. **(A)** A schematic of the tasks undertaken by each cohort of WT and APP/PS1 mice. **(B)** TUNL training and probes were presented in the order they were performed, with samples of the trials seen during each stage of the task displayed. Each trial is shown in the choice phase, thus one square would be the incorrect, previously displayed stimuli while the other would be the correct, newly displayed stimuli. CR, Caloric Restriction; Pre, Pre-training; TUNL, Trial Unique Non-matching to Location; MWM, Morris Water Maze; PR, Progressive Ratio; PAL, Paired Associate Learning; Ind, Individual animal; Grp, Group.

This study was conducted over 3 cohorts of APP/PS1 animals at various ages, which are summarized in Table 1 and Figure 1A. For all experiments, researchers were blind to genotype, with behavioral data automatically collected and analyzed using previously published methods, thus eliminating bias. Group sizes of 7–12 were used across experiments, with the APP/PS1 animals showing a higher premature death rate than their WT littermate counterparts, leading to a smaller number in this group.

**TABLE 1:** Numbers and ages of WT and APP/PS1 animals in each behavioral task.

Animal cohort	Task	Genotype		Age of testing (months)	Age of food restriction (months)	Duration touchscreen testing (months)
		WT	APP/PS1			
Cohort 1	TUNL	9	9	8–11	7–7.25	3
	Y maze	11	9	11.5–12		
	MWM	11	8	11.5–12		
Cohort 2	PR	12	9	8.5–9	7.5–8	7.5
	PAL	12	9	9.5–11.5		
	TUNL	12	8	13-16		
Cohort 3	MWM	7	7	12	N/A	N/A

*This study included three cohorts of animals, ranging in age from 7 to 16 months of age. Animal numbers for each behavioral tasks are broken down by genotype and age groups for each distinct cohort of animals. WT, Wild-type; TUNL, Trial Unique Non-Matching to Location; MWM, Morris Water Maze; PR, Progressive Ratio; PAL, Paired Associate Learning.*

### Behavioral Procedures

#### Touchscreen Pre-training

Pre-training procedures have been described in detail previously (Horner et al., 2013) and occurred before all other tasks. All touchscreen testing was conducted under dim red light during the dark phase of the animals. Animals were initially introduced to the reward (Iced Strawberry Milk, Nippy’s Ltd., Moorook, SA, Australia) in their home cage for 2 days, then habituated to the touchscreen chambers with freely available 7 μL rewards for 20 min for 2 days. Mice were then trained to nose-poke a stimuli for 30 trials on a screen within an hour, for which they were rewarded 7 μL per trial. Following this, animals were required to learn to initiate those 30 trials by placing their head into the food magazine. Finally, animals were punished if they touched the screen anywhere but the stimulus square by a 5 s time out with house light on. During this final stage, animals were required to touch the stimulus rather than the blank parts of the screen for 80% of the 30 trials for 2 consecutive days.

#### Progressive Ratio

Progressive ratio (PR) was performed as previously described (Heath et al., 2015). For this task, animals were first trained on fixed ratio (FR) 2, 3, and 5 paradigms, where the animal must touch the stimuli on the screen 2, 3, or 5 times respectively to receive one reward. Once the animal finished 1 day each of FR2 and FR3, and three consecutive days of FR5, they were moved on to a PR task for 8 days. Here, the number of touches the animal had to make to the screen increased by n + 4 for every reward they collected (e.g., 1 touch, 5 touches, 9, 13, 17 etc.). The breakpoint was calculated as the last full trial completed before the animal stopped responding for 5 min, or the last trial it completed after 1 h.

#### Paired Associate Learning

Paired associate learning (PAL) training was performed as previously described (Horner et al., 2013). PAL tested the ability of the subject to associate an image with a location. In this task, there were three unique images (45°, 145°, and 180° contrast gratings) and each image was only correct in one of three locations, thus the animal was required to learn to associate each image with its corresponding correct location. For each trial, an animal had to first initiate the trial, and was then shown a pair of images, with only one image in the correct location. If the animal chose the correct image, it was rewarded and the next trial presented was a novel trial. If the animal chose incorrectly, the house-light turned on and a 5 s time out was initiated. Following an incorrect trial, the animal was presented the same trial, which was termed a correction trial. The animal was shown the correction trial until it chose the correct image. Correction trials both helped teach the animal the task and gave a measure of how perseverative the animal was (i.e., how willing it was to change from the initial response). Animals could perform up to 36 unique trials per 1-h session, with an unlimited number of correction trials. Animals were trained for 50 days.

#### Trial Unique Non-Matching to Location Training

Trial unique non-matching to location (TUNL) training was performed as previously described (Figure 1B, Kim et al., 2015; Zeleznikow-Johnston et al., 2017a). In the TUNL task, animals were required to touch a square in 1 of 5 locations (sample), then return to the back of the chamber, which triggered the presentation of 2 squares – one in the sample location, and one in a novel location (choice). The animal was rewarded for touching the novel location (thus non-match from the original location). In this task, separation level refers to the number of squares between the sample and choice stimuli, with 3 being the highest level of separation possible and 0 being the lowest. Training took place in two stages. In stage 1, animals were trained to criterion (more than 70% correct for 2 consecutive days) on separation level 3 (S3), separation level 2 (S2) and, finally to separation level 1 (S1). In this stage, S1 trials only consisted of ‘sample’ and ‘choice’ squares in location 2 and 4. Animals were then rested, with weekly reminder sessions, until all animals reached criterion on S1. During stage 1, animals could perform up to 36 trials per 45-min session. Any animals unable to complete this stage of training were excluded (1 APP/PS1 and 2 WT mice). Stage 2 training consisted of 14 days of training on S1 and 4 days of training on separation 0 (S0), with animals able to perform up to 48 trials in 1 h. Here, S1 trials were shown in 1–3 and 3–5 location combinations. During both stages of TUNL training, correction trials were used; i.e., if the animal chose incorrectly, they were shown that same trial repeatedly until they choose the stimuli in the correct, novel location. A schematic of TUNL training is presented in Figure 1B.

#### Trial Unique Non-Matching to Location Pattern Separation and Working Memory Probes

Trial unique non-matching to location can be used to probe both pattern separation and working memory by changing the distance between the sample and choice stimuli and increasing the delay between the sample and choice phase respectively, and procedures to do so have been described previously (Kim et al., 2015; Zeleznikow-Johnston et al., 2017a). Following stage 2 training, all animals underwent the pattern separation probe for 7 days, where trials of S1 and S0 were pseudo-randomly presented during a session, with the S0 trials taxing pattern separation abilities. After completing the pattern separation task, animals were moved onto the working memory probe, where the delay between the sample and choice phase was pseudo-randomly changed between 0, 3, and 6 s, with the longer delays taxing working memory. Animals could perform up to 48 trials in 1 h. Correction trials were disabled for TUNL probes. A schematic of both probes is presented in Figure 1B.

#### Morris Water Maze Training

Morris water maze procedures have been described in detail previously (Rogers et al., 2017). Animals were habituated in the room for 1 day prior to behavioral testing. The pool was divided into quadrants, and each quadrant assigned an ordinal direction. The platform was located in the center of the NE quadrant. Animals were trained for 4 trials per session, once per day for 7 consecutive days. Mice were run in blocks of 6–7 animals per session, with a 15 min inter-trial interval. Within a session, mice were placed under a heat lamp in a standard house cage to prevent hypothermia and allow drying following each trial. The start location was pseudo-randomized using a Latin square design for every trial between NW, W, S, or SE. The animal was allowed to swim around the pool for 60 s to try to find the platform. If the animal did not find the platform in this time, it was gently guided to the platform location by the experimenter. The animal was then required to spend 30 s on the platform with the experimenter out of sight, to allow time for mice to build an allocentric map. Should an animal jump off the platform in that 30 s, it was guided back on and the 30 s would restart. For analysis, average distance to platform for each day of training was assessed.

#### Morris Water Maze Probe Test

On the 8th day, approximately 24 h following the last trial of MWM training, animals underwent a single 2-min probe trial. Here, the platform was removed from the NE quadrant, and animals released into the pool at a novel location (SW). Percentage time spent in each quadrant was analyzed to assess long-term spatial memory for the platform.

#### Discrete-Trial Forced Alternation

Discrete trial forced alternation was performed in the Y maze, as previously described for the T maze (Deacon et al., 2003). Animals were habituated in the room for at least 1 h prior to behavioral testing. At the start of a trial, one of the two proximal arms was blocked off and the animal placed in the home arm facing the wall. The animal was allowed to explore the familiar arm and home arm for 2 min. Animals were then removed from the maze for a 30 s delay, during which the door to the novel arm was removed. The animal was then placed back in the home arm facing the wall, and the initial arm choice (novel or familiar) recorded. The trial ended following the choice or after 2 min with no choice, in which case the animal was removed and ‘no choice’ recorded. Olfactory cues were removed by cleaning between each trial. After an inter-trial interval of 20 min, the animal was run through the same protocol with the opposite ‘familiar’ arm (i.e., if the left arm was initially left open the right arm would be open in the next trial). Each animal was run for 6 ‘choices’ or a max of 8 trials, whichever came first, with the location of the novel arm alternating every trial. Animals with intact spatial memory would be expected to venture into the novel arm for most trials.

### Immunohistochemistry and Quantification

All animals received an intraperitoneal overdose of 80 mg/kg sodium pentobarbital and, when unresponsive, underwent transcardial perfusion with 1x PBS for 2 min. Brains were hemi-sectioned, and the left half dropped fixed in 4% paraformaldehyde overnight, followed by 3 days in 30% sucrose at 4°C and finally freezing with liquid nitrogen for storage at –80°C. For analysis, brains were coronally sectioned (40 μm, 12 series) on a cryostat (Leica), and stored in cryoprotectant (25% ethylene glycol and 25% glycerol) at –20°C until staining. Hippocampal sections were washed in PBS, incubated with 1% H_2_O_2_ for 20 min, blocked with 5% goat serum and finally stained with a rabbit anti-mouse Ki67 or rabbit anti-mouse DCX antibody overnight (1:100, Thermofisher rm9106-s1, 1:200, Abcam ab18723). Following this, sections were incubated with a biotinylated goat anti-rabbit antibody (1:500, Vector BA-1000) and stained with diaminobenzadine chromagen for 15 s. Sections were then mounted on glass slides to dry (DAB) overnight. Finally, slides were dehydrated in ascending alcohol series before being cleared in xylene and coverslipped with DPX mountant (Sigma-Aldrich). Ki67 and DCX counts were conducted by identifying the dentate gyrus (DG) in each section and manually counting the number of DAB positive cells within it. Experimenters were blinded to experimental conditions for counting. Total counts from one series of 12 and both total and average counts per animal (accounting for number of sections/animal) were analyzed, with only total counts presented here for brevity.

### Statistical Approaches

For PR data, an unpaired student’s *t*-test with Welch’s correction was used to compare overall breakpoint. Training effect over days for the PAL and TUNL test were performed with two-way repeated ANOVAs, while number of trials between genotypes were assessed with an unpaired student *t*-test with Welch’s correction with the exception of Stage 1 TUNL trials to criterion, where the log-rank Mantel Cox test was used. For MWM, repeated measures ANOVAs were used to analyze distance to platform and time spent in target quadrant on the probe day. For the effect of genotype and housing on ‘percent novel arm choice’ in the discrete-trial forced alternation and Ki67 + or DCX cell counts, a two-way ANOVA was performed.

PAL, TUNL and discrete-trial forced alternation data was also analyzed at the level of trial (as opposed to animal averages) using generalized linear, latent and mixed regression models (GLLAMM). GLLAMM were used to implement a random effect analysis, with robust standard error estimation and individual animals treated as random effects. These models were used not only as they enable the analysis on a trial-by-trial basis, but also because they work well on non-normally distributed data and can also account for the fact that some animals may be run for a different number of days or may not complete the same number of trials every day. GLLAMM models were run with genotype, day, trial, and, in the case of TUNL, the relevant probe variable (separation level or delay) as explanatory variables in the model. The outcome variables were the odds of correct selection/novel arm selection or the incidence rate ratio of correction trials. Specifically, logistic regressions were used to analyze the binary variables ‘correct selection’ or ‘novel arm selection’ with corresponding effect sizes reported as adjusted odds ratios (aORs) with respective 95% confidence intervals (95% CIs). To interpret these for genotype effects, an aOR of smaller than 1 indicates that APP/PS1 mice are less likely to have chosen correctly, while the corresponding 95% CI not including 1 represents statistical significance (*p* < 0.05). Poisson regressions were used to analyze count data, i.e., the number of correction trials per incorrect trial, with corresponding effect sizes reported as adjusted incidence rate ratios (aIRRs) with respective 95% CIs. The interpretation for aIRR being larger than 1 is that APP/PS1 mice have a higher expected count of correction trials compared to WT animals. For trial-by-trial analysis, odds ratios and incidence rate ratios are presented as effect sizes with a 95% CI All other data is presented as mean ± SEM with individual animals as dots for cross sectional data, or genotype mean ± SEM for longitudinal data. GLLAMM statistical analyses were conducted using STATA v13IC (StataCorp, College Station, TX, United States) with all other analyses being performed on SPSS (IBM Corp, Armonk, NY, United States).

## Results

### APP/PS1 Mice Do Not Show Hippocampal-Dependant Deficits in Paired Associate Learning or Trial Unique Non-Matching to Location Touchscreen Tests

**FIGURE 2:**
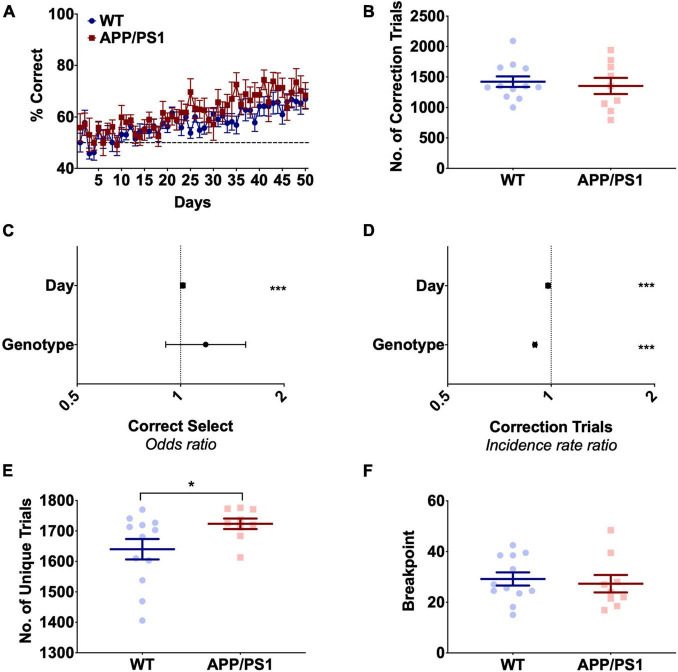
APP/PS1 mice show no deficits in Paired Associate Learning up to 1 year of age. **(A)** Accuracy over 50 days of PAL in 9- to 12-month-old mice, showing no significant effect of genotype. **(B)** APP/PS1 and WT mice performed similar numbers of correction trials during PAL. **(C)** Odds of correct selection in PAL was increased by day but unchanged by genotype. **(D)** Incidence rate ratio of correction trials in PAL was decreased by both day and APP/PS1 genotype. **(E)** APP/PS1 mice performed significantly more unique trials than WT mice over PAL training. **(F)** APP/PS1 and WT mice had similar breakpoints in the progressive ratio task. **(A)** Is presented as group mean ± SEM with over 50 days of training **(B,E,F)** are presented as mean ± SEM with individual animals as dots. **(C,D)** Are the effect size ± 95% CI, showing the effect of genotype and day on odds of correct selection and incidence rate ratio of correction trials. *p < 0.05; ***p < 0.001.

To assess hippocampal function, APP/PS1 mice were assessed on PAL and TUNL between 8 and 16 months of age (Figure 1A). PAL tested the ability of an animal to associate each of the three stimuli shown throughout the task with one of three locations, with each trial showing a pair of images; one in the correct location and one in the incorrect location. Both WT and APP/PS1 animals showed significant improvement on accuracy over the 50 days of PAL training [Figures 2A,C, *F*(49,19) = 6.80, *p* < 0.0001, Supplementary Table 1], with no significant effect of genotype [Figures 2A,C, *F*(1,19) = 1.89, *p* = 0.185, Supplementary Table 1]. Both groups struggled to reach criterion on this task, with both groups averaging only 65–70% correct responses on the final 5 days of training. WT and APP/PS1 mice performed similar numbers of correction trials over the 50 days of training [Figure 2B, *t*(1) = 0.443, *p* = 0.378], but APP/PS1 mice showed a minor but significantly decreased incidence rate ratio of correction trials, equivalent to an APP/PS1 mouse performing 0.9 correction trials for every correction trial a WT mouse performed, with both genotypes showing decreased expected counts of correction trials over days (Figure 2D and Supplementary Table 1). This indicates the APP/PS1 mice in fact performed slightly better than their WT counterparts in this task, directly contrary to our hypothesis. The decreased rate of correction trials in APP/PS1 mice appeared to be driven by an increased number of unique trials performed in this group [Figure 2E, *t*(1) = 2.21, *p* = 0.042]. This increase was not due to differences in motivation, as all animals completing the PAL task initially underwent a PR task and showed equivalent breakpoints [Figure 2F, *t*(1) = 0.434, *p* = 0.67]. The increased number of unique trials performed could be related to the increased perseverative behavior we have previously shown in APP/PS1 mice during difficult touchscreen tasks (Shepherd et al., 2019). Due to the low level of both performance and engagement with this task, we opted to assess hippocampal function using the TUNL test.

The TUNL task tested both pattern separation and working memory, by requiring animals to remember the previous location of a stimuli (when shown a pair of stimuli) by picking the stimulus in the novel location (thereby ‘non-matching’ with the previous single stimulus, Figure 1B). The number of squares between the sample and choice square was termed separation level, and as separation level decreased throughout training, task difficulty was increased (Figure 1B). Both touchscreen cohorts of animals from this study underwent TUNL, starting at 8 or 13 months of age (Figure 1A). There was no effect of genotype on Stage 1 training in the 8-month-old [Supplementary Figure 1a, χ2(1) = 0.006, *p* = 0.938] or 13-month-old animals [Supplementary Figure 1b, χ2(1) = 0.467, *p* = 0.494]. When assessing the data at the trial level, there was no effect of day, genotype, or separation level on the odds of an animal correctly selecting the rewarded image in the younger or older cohort (Supplementary Figures 1c,d and Supplementary Table 1). The lack of effect of day, genotype or separation level concords with the null hypothesis that for any given trial on any given day or separation level, WT and APP/PS1 animals of both ages had the same likelihood of choosing the correct stimuli. This probably reflects the fact that animals were trained to criterion on this task. The number of correction trials was unaffected by genotype in 8-month-old [Supplementary Figure 1e, *t*(1) = 0.922, *p* = 0.383] or 13-month-old [Supplementary Figure 1f, *t*(1) = 0.691, *p* = 0.5] mice. When assessing the expected count of correction trials per trial in the 8-month-old mice, there was no effect of day, genotype or separation level on the incidence rate ratio of correction trials (Supplementary Figure 1g and Supplementary Table 1). Similarly, there was also no effect of day, genotype or separation level on the expected count of correction trials in the 13-month-old mice (Supplementary Figure 1h and Supplementary Table 1). This indicated that WT and APP/PS1 mice at both ages performed a similar number of correction trials per trial, regardless of what day of training they are on, or the separation level of the trial. In the first cohort of animals, three mice did not reach criterion (two WT and one APP/PS1 mice), while in the second cohort of animals all animals reached criterion. All animals that did not reach criterion were removed from analysis.

Following completion of stage 1 training, animals were moved to stage 2 training, where they were trained for 14 days on separation level one (S1) and 4 days on separation level zero (S0), where there was 1 gap or 0 gaps between the pair of stimuli presented respectively (Figure 1B). Animals showed decreased accuracy on S0 compared to S1 in both the now 10-month-old [Supplementary Figures 2a,c, *F*(1,18) = 6.65, *p* < 0.0001, Supplementary Table 1] and 15-month-old cohorts [Supplementary Figures 2b,d, *F*(1,18) = 4.43, *p* < 0.0001, Supplementary Table 1], indicating that animals found S0 more difficult than S1, as expected. APP/PS1 mice were as accurate both overall and at the trial level as WT mice in both the 10-month-old [Supplementary Figures 2a,c, *F*(1,18) = 0.028, *p* = 0.869, Supplementary Table 1] or 15-month-old groups [Supplementary Figures 2b,d, *F*(1,18) = 0.040, *p* = 0.843, Supplementary Table 1]. The odds of correct selection were unaffected in 10-month-old animals over time (Supplementary Figure 2c and Supplementary Table 1), while the odds of correct selection increased in 15-month-old animals (Supplementary Figure 2d and Supplementary Table 1). This indicated that while the 10-month-old mice performed similarly across stage 2 training, 15-month-old animals were more likely to choose the correct stimulus (when accounting for separation level) at the end of the 14-day training period. As animals performed fewer days at S0, the number of correction trials performed during stage 2 was significantly decreased by separation level in the younger and older cohorts (Supplementary Figures 2e,f and Supplementary Table 1), as expected. APP/PS1 performed the same number of correction trials as their WT counterparts in both the 10-month-old and 15-month-old cohorts at both separation levels (Supplementary Figures 2e,f and Supplementary Table 1). When assessing the expected count of correction trials per trial, both day and separation level significantly decreased the expected count of correction trials performed in 10-month-old mice and 15-month-old mice (Supplementary Figures 2g,h and Supplementary Table 1). This indicated that animals performed fewer correction trials every consecutive day over training, and also that they performed less correction trials per trial on S1 than S0 trials. APP/PS1 mice had equivalent expected count of correction trials compared to WT mice in both the 10-month-old and 15-month-old animals (Supplementary Figures 2g,h and Supplementary Table 1). In the first cohort of animals, one mouse did not complete stage 2 training due to a calculation error, and was excluded.

**FIGURE 3:**
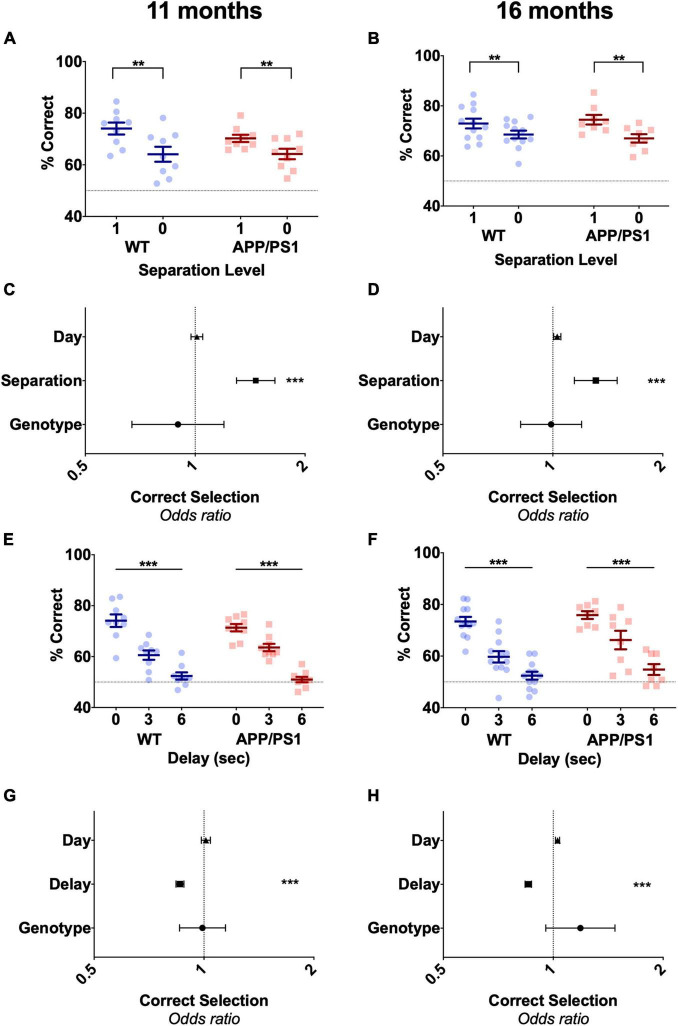
Both 11-month-old and 16-month-old APP/PS1 mice do not show pattern separation or spatial working memory deficits. **(A)** Accuracy on both S1 and S0 trials in 11-month-old mice or **(B)** 16-month-old mice was unaffected by genotype but decreased by separation level in both groups. **(C)** Odds of correct selection was unaffected by day and genotype but increased by increasing separation level in 11-month-old mice and **(D)** 16-month-old mice. **(E)** Similarly, genotype had no effect on accuracy over a 0, 3, and 6-s delay in 11-month-old mice or **(F)** 16-month-old mice, although longer delays significantly decreased accuracy in both ages. **(G)** Odds of correct selection was unaffected by day and genotype but decreased by delay in 11-month-old mice and **(H)** 16-month-old mice. **(A,B,E,F)** Are presented as mean ± SEM with individual animals as dots. **(C,D,G,H)** Are the effect size ± 95% CI, showing the effect of genotype, day and separation level or delay on odds of correct selection. Dotted line in **(A,B,E,F)** represents chance performance (50%). **p < 0.01; ***p < 0.001.

Following stage 2 training, animals undertook 7 days of the pattern separation probe, where S1 and S0 trials were presented variably within the session. As expected, animals showed decreased accuracy during S0 trials at the overall level in both 11-month-old [Figure 3A, *F*(1,18) = 50.5, *p* < 0.0001] and 16-month-old animals [Figure 3B, *F*(1,18) = 18.43, *p* = 0.0004]. When scrutinized at the level of trial, separation level was the only factor that affected in the odds of correct selection in both 10 and 15 months old mice (Figures 3C,D and Supplementary Table 1). No differences in performance due to genotype during S0 trials were observed, with similar accuracy both overall or at the level of trial for APP/PS1 and WT mice in both 11 months old [Figures 3A,C, *F*(1,18) = 0.395, *p* = 0.539, Supplementary Table 1] and 16 months old [Figures 3B,D, *F*(1,18) = 0.00002, *p* = 0.996, Supplementary Table 1]. Day also had no effect on the odds of correct selection at either age (Figures 3C,D and Supplementary Table 1). This indicated both WT and APP/PS1 were equally likely to select the correct stimuli on every day of the pattern separation probe. Following the pattern separation probe, animals undertook the working memory probe. Here, separation level 1 was maintained, but the delay between the sample (1 square) and choice (2 square) phase was increased to 3 or 6 s to tax working memory, or kept at 0 as a baseline measure. As the delay increased, the overall accuracy of response in 11-month-old [Figure 3E, *F*(2,18) = 96.3, *p* < 0.0001] and 16-month-old [Figure 3F, *F*(2,18) = 81.61, *p* < 0.001] animals significantly decreased, indicating that longer delays are more difficult for animals to perform. APP/PS1 mice showed similar accuracy to WT mice at the three different delays in both 11-month-old [Figure 3E, *F*(2,18) = 0.059, *p* = 0.8119] and 16-month-old animals [Figure 3F, *F*(2,18) = 2.433, *p* = 0.1362]. When investigating this data at the level of trial, only increasing delay significantly decreased the odds of correct selection in both 11-month-old and 16-month-old animals (Figures 3G,H and Supplementary Table 1). Neither genotype nor day was found to have a statistically significant effect on the odds of correct selection in the 11-month-old mice or in the 16-month-old cohort of animals (Figures 3G,H and Supplementary Table 1), indicating that APP/PS1 and WT mice were just as likely to choose the correct stimuli as each other on any day of the working memory probe. Thus, APP/PS1 did not show any hippocampal dependant deficits up to 16 months of age on the TUNL task and its probes. This was surprising, as APP/PS1 mice commonly show deficits on the hippocampal dependant MWM by 12 months of age (Jankowsky et al., 2005; Hooijmans et al., 2009; Guo et al., 2015; Tapia-Rojas et al., 2016).

### Touchscreen Testing Increases Neurogenesis Markers and Improves Performance on the Morris Water Maze

**FIGURE 4:**
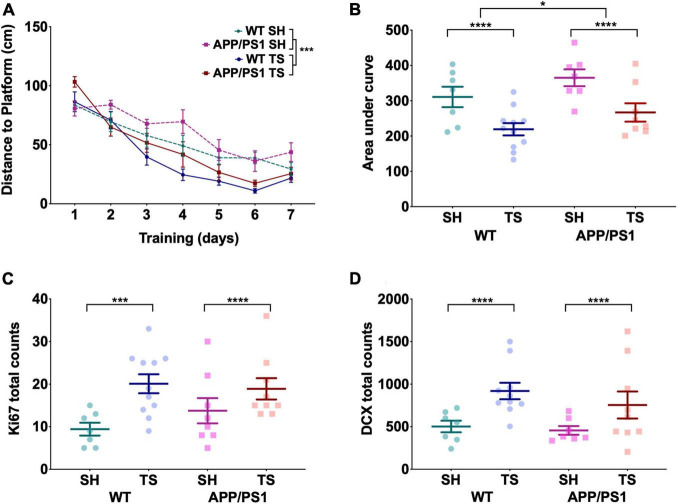
Touchscreen training improved cognitive ability in the MWM and increased the number of immature neurons and proliferating cells in the dentate gyrus. **(A)** The average distance to platform of the 7 days of MWM training. A significant decrease in distance was seen over the days, with touchscreen-trained animals showing significantly shorter distances to platform regardless of genotype. **(B)** Touchscreen tested (TS) animals showed a significantly smaller area under the curve of the 7 days of MWM training, while APP/PS1 mice showed significantly larger AUC than their WT counterparts. **(C)** Total count of Ki67 + cells seen throughout the dentate gyrus, with no effect of genotype but TS tested animals showed significantly more Ki67 + cells than standard housed (SH) animals. **(D)** Total count of DCX + cells seen throughout the dentate gyrus, where TS mice showed significantly more DCX + cells than SH animals and genotype had no effect. **(A)** Is presented as mean ± SEM with the average distance for each group on a given day shown as a dot. **(B–D)** Are presented as mean ± SEM with individual animals as dots. *p < 0.05; ***p < 0.001; ****p < 0.0001.

To test if we could recapitulate the reported hippocampal-dependant MWM deficit at 12 months in our APP/PS1 mice, we trained both the first TUNL tested cohort and behaviorally naïve, standard-housed 12-month-old APP/PS1 and WT mice on the MWM as well as a discrete-trial forced alternation task in a Y-maze (Figure 1A). Over the 7 days of MWM training, the distance to platform significantly reduced with no day-by-genotype or day-by-touchscreen exposure interaction effects, indicating learning in all groups [Figure 4A, *F*(6,24) = 59.22, *p* < 0.0001]. However, there was a significant effect of prior touchscreen training on distance to platform, with touchscreen-trained animals showing shorter distances to platform [Figure 4A, *F*(1,29) = 61.70, *p* < 0.0001] with no effect of genotype [Figure 4A, *F*(1,29) = 1.74, *p* = 0.20]. When assessing the area under the curve (AUC), there was a small but significant effect of genotype [Figure 4B, *F*(1,3) = 4.61, *p* = 0.04], with APP/PS1 mice showing larger AUC in both SH and TS groups, although no group was significantly different following *post hoc* testing. However, this genotype difference paled in comparison to the effect of prior TUNL training [Figure 4B, *F*(1,3) = 15.98, *p* < 0.0001] with the WT and APP/PS1 touchscreen-tested groups showing significantly smaller AUC than both standard-housed groups. There was no interaction effect between genotype and housing [*F*(1,3) = 0.020, *p* = 0.89]. Touchscreen-trained groups showed increased swimming velocity across all days of training except day 3 and 6 compared to their standard-housed counterparts, regardless of genotype [Supplementary Figure 3a, *F*(6,24) = 2.67, *p* = 0.040], which is why we chose to analyze distance rather than latency to platform. This is unlikely to be driven by better learning in the touchscreen group, as velocity was significantly different on day 1 and remained stable across training days. There was no effect of genotype on velocity [Supplementary Figure 3a, *F*(6,24) = 0.643, *p* = 0.7]. All groups showed a significant preference for the target quadrant during the MWM probe trial [Supplementary Figure 3c, *F*(3,27) = 16.42, *p* < 0.0001], with touchscreen-trained animals showing a stronger preference than standard-housed animals [Supplementary Figure 3b, *F*(1,29) = 44.62, *p* < 0.0001], with no effect of genotype [Supplementary Figure 3b, *F*(1,29) = 0.51, *p* = 0.82] nor genotype by prior-touchscreen-training interaction effects [Supplementary Figure 3b, *F*(1,29) = 0.012, *p* = 0.91]. All groups performed extremely well on the discrete-trial forced-alternation task (Supplementary Figure 3c), with an average accuracy of 75.86% and no effect of touchscreen testing [Supplementary Figures 3c,d, *F*(1,3) = 0.22, *p* = 0.883] or genotype [Supplementary Figures 3c,d, *F*(1,3) = 0.12, *p* = 0.914]; this is possibly due to a ceiling effect, as average performance was between 73.8 and 78.8% across all groups.

This marked effect of touchscreen training on MWM performance led us to examine the possibility of increased hippocampal neurogenesis in our touchscreen trained mice. We assessed levels of proliferation and numbers of immature neurons in the dentate gyrus using Ki67 and doublecortin (DCX) respectively. These measures have previously been shown to be good proxy measures of neurogenesis in this region of the hippocampus (Kee et al., 2002). Touchscreen-tested WT and APP/PS1 mice showed elevated levels of proliferation [Figure 4C, *F*(1,5) = 10.633, *p* = 0.002] and immature neurons [Figure 4D, *F*(1,5) = 14.854, *p* < 0.0001] compared to standard-housed animals, indicating that touchscreen training may increase hippocampal neurogenesis. There was no effect of genotype on number of proliferating cells [Figure 4C, *F*(1,5) = 0.607, *p* = 0.440] or immature neurons [Figure 4D, *F*(1,5) = 1.803, *p* = 0.186], indicating that the effect of touchscreen training was equal in both WT and APP/PS1 mice. Representative images of Ki67 and DCX staining can be found in Supplementary Figures 4c,d.

We also assessed if this increase would be sustained following the cessation of touchscreen training. The cohort that finished TUNL at 16 months of age were rested for 5 months until 21 months of age, and had the number of Ki67 + and DCX + in the dentate gyrus quantified following this rest period. 21-month-old animals had significantly less Ki67 + cells [Supplementary Figures 4a,c, *F*(1,5) = 28.48, *p* < 0.0001] and DCX + cells [Supplementary Figures 4b,d, *F*(1,5) = 59.77, *p* < 0.0001] than both 12 months groups, indicating that touchscreen testing benefits are not fully sustained following touchscreen training. Unfortunately, we did not have access to an age-matched, behaviorally naive cohort, so it is unclear if the touchscreen testing benefit is only partially or completely lost as the animals age.

## Discussion

This is the first study to show evidence that touchscreen testing can act as a cognitive enhancer that boosts long-term memory task acquisition and the number of proliferating cells and immature neurons in the hippocampus. No overt differences in accuracy in either the PAL or TUNL tasks were seen between APP/PS1 and WT mice. When mice previously trained on touchscreen tasks for 4 months were assessed on the MWM, all touchscreen mice, regardless of genotype, showed improved MWM acquisition compared to training-naïve mice. This effect was not seen on a short-term memory task. Overall, APP/PS1 mice did show slightly slowed learning in the water maze compared to WT mice, with an unchanged preference for the target quadrant during the memory probe. Exposure to touchscreen training in 12-month-old mice increased the number of cells positive for Ki67, a cell proliferation marker and DCX, a marker of immature neurons.

It was surprising to find intact cognition in 8–16 months old APP/PS1 mice using TUNL and PAL touchscreen tasks, as cognitive deficits have been reported in this model as early as 4 months of age (Bonardi et al., 2011). Furthermore, while APP/PS1 mice showed delayed learning in the MWM, this did not result in a recall impairment during the probe trial. Many reports of MWM impairments in both task acquisition and memory recall in APP/PS1 mice exist, however unimpaired MWM performance has also been shown in APP/PS1 mice at 5–8 as well as 20–26 months of age (Stover and Brown, 2012). This phenomenon, with identical mouse models showing drastically different timing of symptom-onset, with some deficits occurring in a subset of facilities and not in others, is a well-known problem with transgenic AD models (Stewart et al., 2011), as well as preclinical models of a wide variety of other disorders. Traditional cognitive tests are inherently more sensitive to extraneous confounds due to the short nature of training/assessment and with publication bias favoring studies showing impairments, the balance of evidence may be skewed. As an example, we have previously documented impairments in executive function in APP/PS1 mice using touchscreen tasks (Shepherd et al., 2021), however, traditional tasks have seen executive function deficits at a much younger age (Jankowsky et al., 2005; Filali and Lalonde, 2009; Hooijmans et al., 2009; Filali et al., 2011; Stover and Brown, 2012). Another possible explanation for the milder impairment reported in APP/PS1 mice in our study is the inclusion of only male mice. Female APP/PS1 mice have a notably divergent disease course compared to male mice, with accelerated onset of cognitive changes and Aβ deposition but improved lifespan (Rae and Brown, 2015; Jankowsky and Zheng, 2017). Some studies have shown deficits solely in female mice (Gallagher et al., 2013); however, the vast majority do see MWM deficits in male mice too (Jankowsky et al., 2005; Savonenko et al., 2005; Cao et al., 2007; Hooijmans et al., 2009; Stover and Brown, 2012; Guo et al., 2015; Jiao et al., 2015), so this is not the only explanation for our mild phenotype. Traditional maze-based tasks often rely on stressful situations to motivate behavior. Stress is known to drive AD progression in humans and animals models (reviewed in Justice, 2018) and can decrease cognitive performance in both WT (reviewed in Moreira et al., 2016) and APP/PS1 mice (Han et al., 2016, 2017); an interaction between AD pathology, stress and phenotype is likely. Studies in support of a stress interaction include reports of extinction impairments in APP/PS1 mice, which were only reported in aversive extinction paradigms (Ramírez-Lugo et al., 2009; Bonardi et al., 2011; Cheng et al., 2019), but not appetitive extinction paradigms (Bonardi et al., 2011). It is possible that the stress induced by these paradigms could account for the deficits seen in much younger APP/PS1 mice, compared to those tested in our rewarded touchscreen paradigm.

We reported increased cell proliferation and immature neurons in the dentate gyrus of WT and APP/PS1 mice following 3 months of touchscreen training. Increased neurogenesis is a well-described outcome from exercise and environmental enrichment interventions, and may indicate that touchscreen testing has a similar effect on the brain as these two interventions. While numbers of proliferating cells and immature neurons have been shown previously to highly correlate with the number of cells incorporating DNA synthesis markers like BrdU in the hippocampus (Kee et al., 2002), this remains to be confirmed in our study. Reduced neurogenesis has been previously been observed in APP/PS1 mice at 9 months of age (Taniuchi et al., 2007) and this result also suggests that long-term touchscreen training has the potential to not only promote neurogenesis in WT mice, but could also rescue neurogenesis deficits in AD mouse models, and thus have the potential to rescue cognitive deficits (reviewed in Shepherd et al., 2018). This was unable to be directly tested in this study, as APP/PS1 mice at 12 months showed similar Ki67+ and DCX+ neurons to their wildtype counterparts, and should be evaluated in future studies. This finding is critically important for the design and analysis of behavior obtained using touchscreen training, especially in the context of models in which hippocampal neurogenesis may be affected, such as AD models.

What aspects of the touchscreen testing paradigm are driving neurogenesis and improved cognition? It could be that extended touchscreen testing increases brain and cognitive reserve (reviewed in Zeleznikow-Johnston et al., 2017b), akin to the broad effects seen following environmental enrichment and exercise paradigms (reviewed in Shepherd et al., 2018). Our study is not the first to investigate the ability of touchscreen-based training to induce changes in hippocampal neuronal activity in mice. Mice trained on a cognitive flexibility touchscreen paradigm showed upregulated immediate-early gene induction in the hippocampus and increased BDNF gene expression (Mallien et al., 2016). Upregulation of BDNF has been shown to be central to EE-induced increases in neurogenesis (Nithianantharajah and Hannan, 2009). To our knowledge, no other study has investigated the cognitive enhancing effects of touchscreen training, nor shown improved behavioral performance following touchscreen training as seen in this study. Both of the touchscreen tasks used in this study, PAL and TUNL, assess aspects of hippocampal-dependent cognition; and thus acquiring these tasks could result in transference to other tasks requiring similar brain regions (i.e., MWM). It has been noted that cognitive gains in humans undergoing CCT are most related to the domain being specifically trained – i.e., that extensive training with associative learning may not generalize to improved performance on attention tasks, and vice versa (Lampit et al., 2014). In support of this, preclinical studies over-training AD animals on the MWM have shown improvements in other hippocampal-dependent tasks, namely novel-object recognition and fear conditioning (Billings et al., 2007; Jiang et al., 2015; Martinez-Coria et al., 2015), supporting the idea that undergoing cognitive tasks can improve cognitive performance in animals as well as humans. Our study was unable to determine whether the transference effect seen with touchscreen training is domain specific, thus future studies could compare mice trained on hippocampal vs. non-hippocampal tasks to test the specificity of these effects.

Another possible explanation for the observed improvement in cognitive performance and increased neurogenesis is CR. CR is an inherent feature of touchscreen testing paradigm, as animals must be food-restricted to motivate performance in the cognitive tasks. However, CR itself has a range of positive effects on both WT mice and AD mouse models. In WT mice, CR increases neurogenesis in the hippocampus, increases synaptic plasticity markers and neurotrophic growth factors across the brain, while also decreasing age-induced increases in stress and inflammation genes (Lee et al., 2000, 2002). Furthermore, CR can rescue age-related deficits in learning and memory tasks (reviewed in Van Cauwenberghe et al., 2016). In the context of AD models, CR can decrease Aβ load in APP/PS1 mice (Mouton et al., 2009) as well as many other AD mouse models (Patel et al., 2005; Wang et al., 2005; Halagappa et al., 2007; Dhurandhar et al., 2013; Schafer et al., 2015). Of note, 30% CR and intermittent fasting have both been shown to rescue MWM performance in 17-month-old 3xTG-AD mice (Halagappa et al., 2007). The effect of CR on neurogenesis in AD models has not been previously investigated. Touchscreen-trained animals also engage in increased incidental exercise during touchscreen training and, combined with CR, this could account for increased velocity observed in touchscreen-trained animals in the MWM. It is difficult to dissociate the effects of CR from touchscreen training without a CR-only control group. A study designed to elucidate the specific contributions of CR, increases in incidental exercise, reward and enhanced cognitive stimulus that result from touchscreen training will be critical to understanding how the touchscreen paradigm can affect animals undergoing such training.

As the touchscreen testing paradigm increases in prevalence as the preferred method for assessing cognition in rodent models (Dumont et al., 2020), it is critical to understand how this intervention may be affecting the brain and behavior of the animals undergoing the training, to ensure that the potential confounds of touchscreen training are accounted for when making conclusions. While this may make design and analysis of long-term studies more complicated, the fact that touchscreen training may affect the brain in a way analogous to environmental enrichment could be considered a benefit. Firstly, standard-housed laboratory animals that receive no cognitive stimulation throughout their lives are not likely to be representative of people who are cognitive challenged throughout their lives (Burrows and Hannan, 2013); interventions that are effective on top of the cognitively enhancing effects of touchscreen training may be more likely to work in human patients. Secondly, touchscreen testing may provide a preclinical model of CCT, which is one of few interventions that can ameliorate symptoms in MCI patients (Hill et al., 2017). A preclinical model of CCT will allow elucidation of the molecular effects of this intervention and lead to novel drug targets for both MCI and AD. Future studies should focus on delineating the effects of CR, exercise, novel environment exposure and cognitive stimulation to better understand the effect of touchscreen training, so as to delineate the molecular, cellular and systemic impacts of touchscreen-based cognitive stimulation. The fact that touchscreen training improves cognition provides important context for future touchscreen studies where hippocampal dysfunction may be rescued by the touchscreen-training paradigm, and opens up exciting opportunities to understand how interventions like CCT may be effective in MCI and other neurological and psychiatric disorders.

## Data Availability Statement

The original contributions presented in the study are included in the article/Supplementary Material, further inquiries can be directed to the corresponding author/s.

## Ethics Statement

The animal study was reviewed and approved by Florey Institute of Neuroscience and Mental Health Animal Ethics Committee.

## Author Contributions

AS: conceptualization, methodology, formal analysis, investigation, writing – original draft, visualization, and writing – review and editing. TZ: methodology, formal analysis, investigation, writing – original draft, visualization, and writing – review and editing. LBH: methodology, and review and editing. AZ-J: methodology, software, formal analysis, writing – original draft, and writing – review and editing. LC: methodology, and writing – review and editing, supervision. AH: resources, and writing – review and editing, supervision. EB: conceptualization, methodology, formal analysis, investigation, writing – original draft, resources, writing – review and editing, and supervision, project administration, and funding acquisition. All authors contributed to the article and approved the submitted version.

## Conflict of Interest

The authors declare that the research was conducted in the absence of any commercial or financial relationships that could be construed as a potential conflict of interest.

## Publisher’s Note

All claims expressed in this article are solely those of the authors and do not necessarily represent those of their affiliated organizations, or those of the publisher, the editors and the reviewers. Any product that may be evaluated in this article, or claim that may be made by its manufacturer, is not guaranteed or endorsed by the publisher.

## Supplementary Material

Supplementary Figure 1 APP/PS1 mice show no differences in Stage 1 training in TUNL at 8 or 13 months of age. **(a)** Genotype had no effect on the number of trials to criterion for TUNL stage 1 training in 8-month-old mice or **(b)** 13-month-old mice. **(c)** Odds of correct selection was unaffected by day, separation level and genotype in 8-month-old mice or **(d)** 13-month-old mice. **(e)** Genotype had no effect on the number of correction trials to criterion for TUNL Stage 1 training in 8-month-old mice or **(f)** 13-month-old mice. **(g)** There was no effect of genotype, day and separation level on the incidence rate ratio of correction trials in 8-month-old mice or **(h)** 13-month-old mice. **(a,b)** Are presented as survival curves, with each ‘step’ on the curve representing 1 or more animals. **(e,f)** Are presented as mean ± SEM with individual animals as dots. **(c,d,g,h)** Are the effect size ± 95% CI, showing the effect of genotype, day and separation level on odds of correct selection and incidence rate ratio of correction trials respectively.

Supplementary Figure 2 APP/PS1 mice show no differences in Stage 2 training in TUNL at 10 or 15 months of age. **(a)** Genotype had no effect on accuracy over TUNL stage 2 training in 10-month-old mice or **(b)** 15-month-old mice. **(c)** Odds of correct selection was unaffected by day and genotype but increased by separation level in 10-month-old mice and **(d)** and increased by day and separation level in 15-month-old mice. **(e)** Genotype had no effect on the number of correction trials performed over the two separation levels of TUNL stage 2 training in 10-month-old mice or **(f)** 15-month-old mice. **(g)** There was no effect of genotype on the incidence rate ratio of correction trials with 10-month-old mice or **(h)** 15-month-old mice, while as day and separation level increased, the incidence rate ratio of correction trials decreased in both ages. **(a,b)** Is presented as group mean ± SEM over 18 days of training. **(e,f)** Are presented as mean ± SEM with individual animals as dots. **(c,d,g,h)** Are the effect size ± 95% CI, showing the effect of genotype, day and separation level on odds of correct selection and incidence rate ratio of correction trials. **p* < 0.05; ^***^*p* < 0.001; ^****^*p* < 0.0001.

Supplementary Figure 3 Touchscreen-tested mice swim more quickly and show a stronger target quadrant preference than their standard housed counterparts in the MWM. **(a)** Both WT and APP/PS1 touchscreen-trained mice showed a higher swimming velocity in the MWM training across all days excepting day 3 and 6. **(b)** All groups showed a preference for the target quadrant during the MWM probe trial, with both WT and APP/PS1 touchscreen-trained groups showing a stronger preference than their standard-housed, behaviorally naïve counterparts. **(c)** All groups showed a strong preference for the novel arm during a discrete trial forced alternation task. **(d)** Neither genotype nor prior touchscreen exposure altered the odds of selecting the novel arm during discrete trial forced alternation. **(a)** Is presented as group mean ± SEM over 7 days of training. **(b,c)** Are presented as mean ± SEM with individual animals as dots. **(d)** Is the effect size ± 95% CI, showing the effect of genotype and prior touchscreen exposure on odds of novel arm selection. Dotted line in **(b,c)** represents chance performance (50%). **p* < 0.05; ^****^*p* < 0.0001.

Supplementary Figure 4 High numbers of proliferating cells and immature neurons are not maintained in mice rested after completing touchscreen testing. **(a)** Total Ki67 counts are elevated following touchscreen training (TS) in both WT and APP/PS1 mice compared to standard housed (SH) animals, but this effect is lost when animals are rested (R) for 5 months following touchscreen training. **(b)** Total DCX counts are elevated following touchscreen training in both WT and APP/PS1 mice compared to SH animals, but this effect is lost when animals are rested for 5 months following touchscreen training. **(c)** Representative images of Ki67 DAB staining in WT and APP/PS1 mice that were SH, underwent TS training or were R following touchscreen training. **(d)** Representative images of DCX DAB staining in WT and APP/PS1 mice that were SH, underwent TS training or were R following touchscreen training. **(a,b)** Are presented as mean ± SEM with individual animals as dots. ^****^*p* < 0.0001.
