# Supplementary figures and images for: A Preclinical Model of Computerized Cognitive Training: Touchscreen Cognitive Testing Enhances Cognition and Hippocampal Cellular Plasticity in Wildtype and Alzheimer’s Disease Mice
Source: Front Behav Neurosci. 2021 Dec 6;15:766745. doi: 10.3389/fnbeh.2021.766745 (PMC8685297; doi:10.3389/fnbeh.2021.766745)

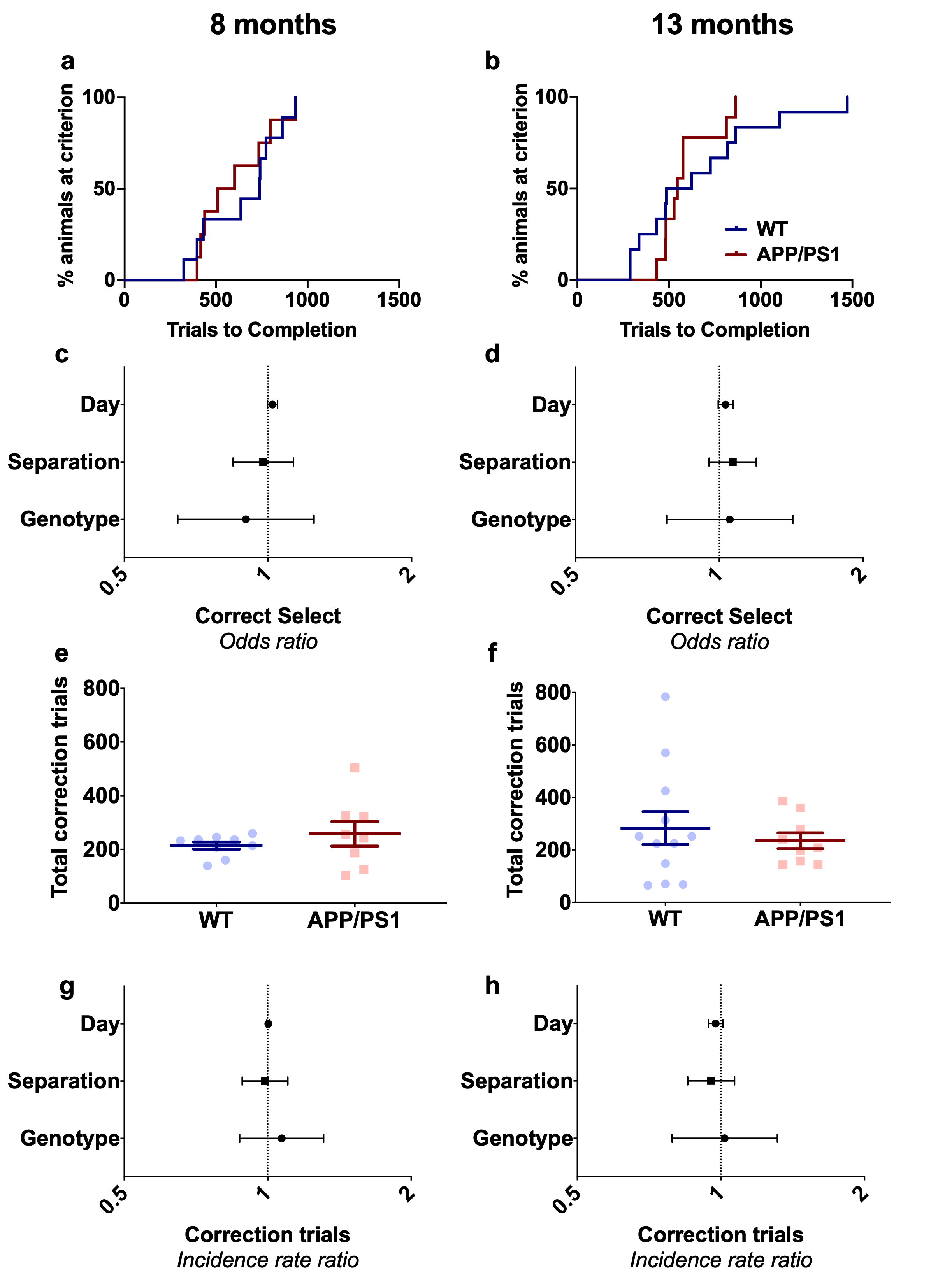

Supplement: Supplementary Figure 1 — APP/PS1 mice show no differences in Stage 1 training in TUNL at 8 or 13 months of age. (a) Genotype had no effect on the number of trials to criterion for TUNL stage 1 training in 8-month-old mice or (b) 13-month-old mice. (c) Odds of correct selection was unaffected by day, separation level and genotype in 8-month-old mice or (d) 13-month-old mice. (e) Genotype had no effect on the number of correction trials to criterion for TUNL Stage 1 training in 8-month-old mice or (f) 13-month-old mice. (g) There was no effect of genotype, day and separation level on the incidence rate ratio of correction trials in 8-month-old mice or (h) 13-month-old mice. (a,b) Are presented as survival curves, with each ‘step’ on the curve representing 1 or more animals. (e,f) Are presented as mean ± SEM with individual animals as dots. (c,d,g,h) Are the effect size ± 95% CI, showing the effect of genotype, day and separation level on odds of correct selection and incidence rate ratio of correction trials respectively. [file Image_1.TIFF]

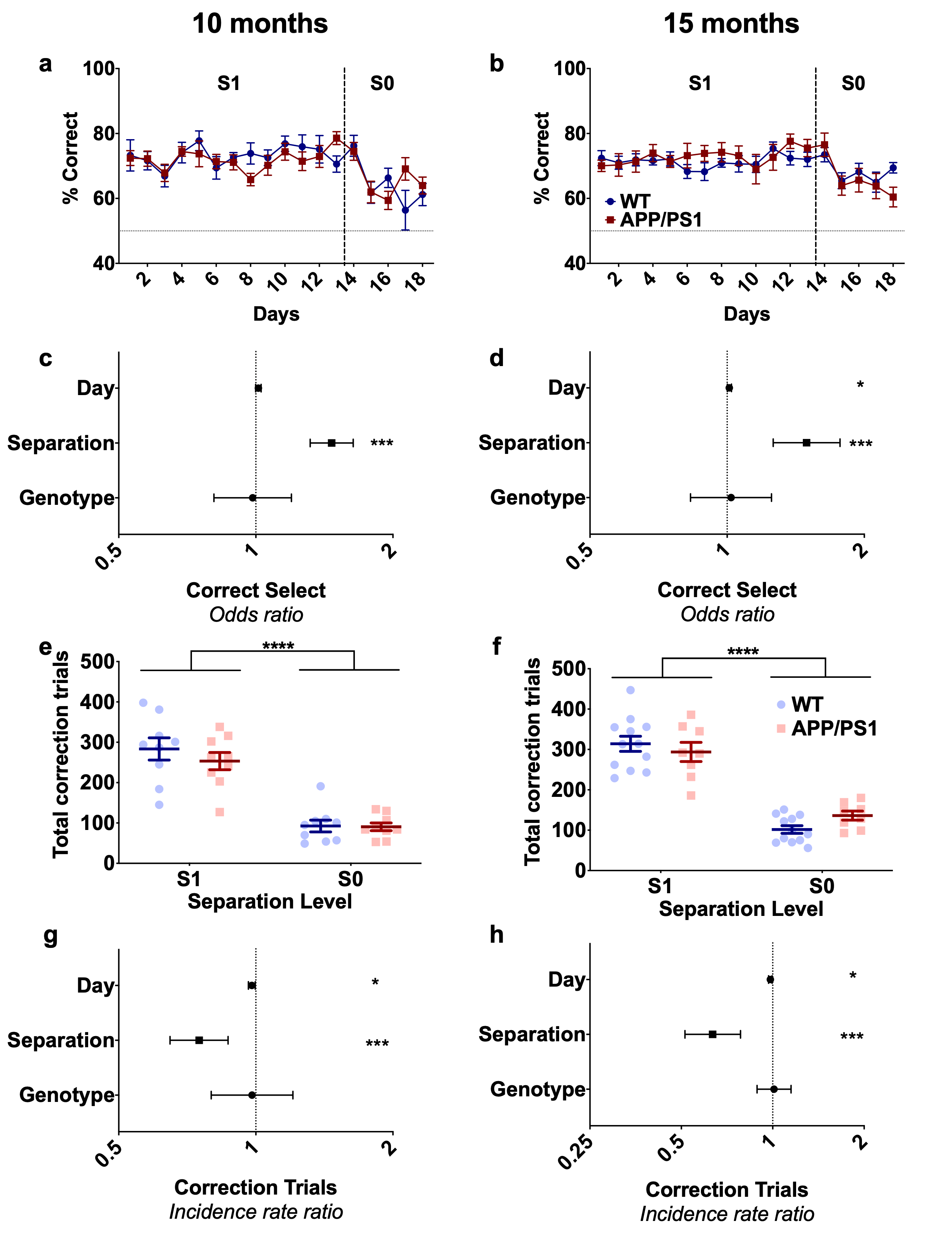

Supplement: Supplementary Figure 2 — APP/PS1 mice show no differences in Stage 2 training in TUNL at 10 or 15 months of age. (a) Genotype had no effect on accuracy over TUNL stage 2 training in 10-month-old mice or (b) 15-month-old mice. (c) Odds of correct selection was unaffected by day and genotype but increased by separation level in 10-month-old mice and (d) and increased by day and separation level in 15-month-old mice. (e) Genotype had no effect on the number of correction trials performed over the two separation levels of TUNL stage 2 training in 10-month-old mice or (f) 15-month-old mice. (g) There was no effect of genotype on the incidence rate ratio of correction trials with 10-month-old mice or (h) 15-month-old mice, while as day and separation level increased, the incidence rate ratio of correction trials decreased in both ages. (a,b) Is presented as group mean ± SEM over 18 days of training. (e,f) Are presented as mean ± SEM with individual animals as dots. (c,d,g,h) Are the effect size ± 95% CI, showing the effect of genotype, day and separation level on odds of correct selection and incidence rate ratio of correction trials. *p < 0.05; ***p < 0.001; ****p < 0.0001. [file Image_2.TIFF]

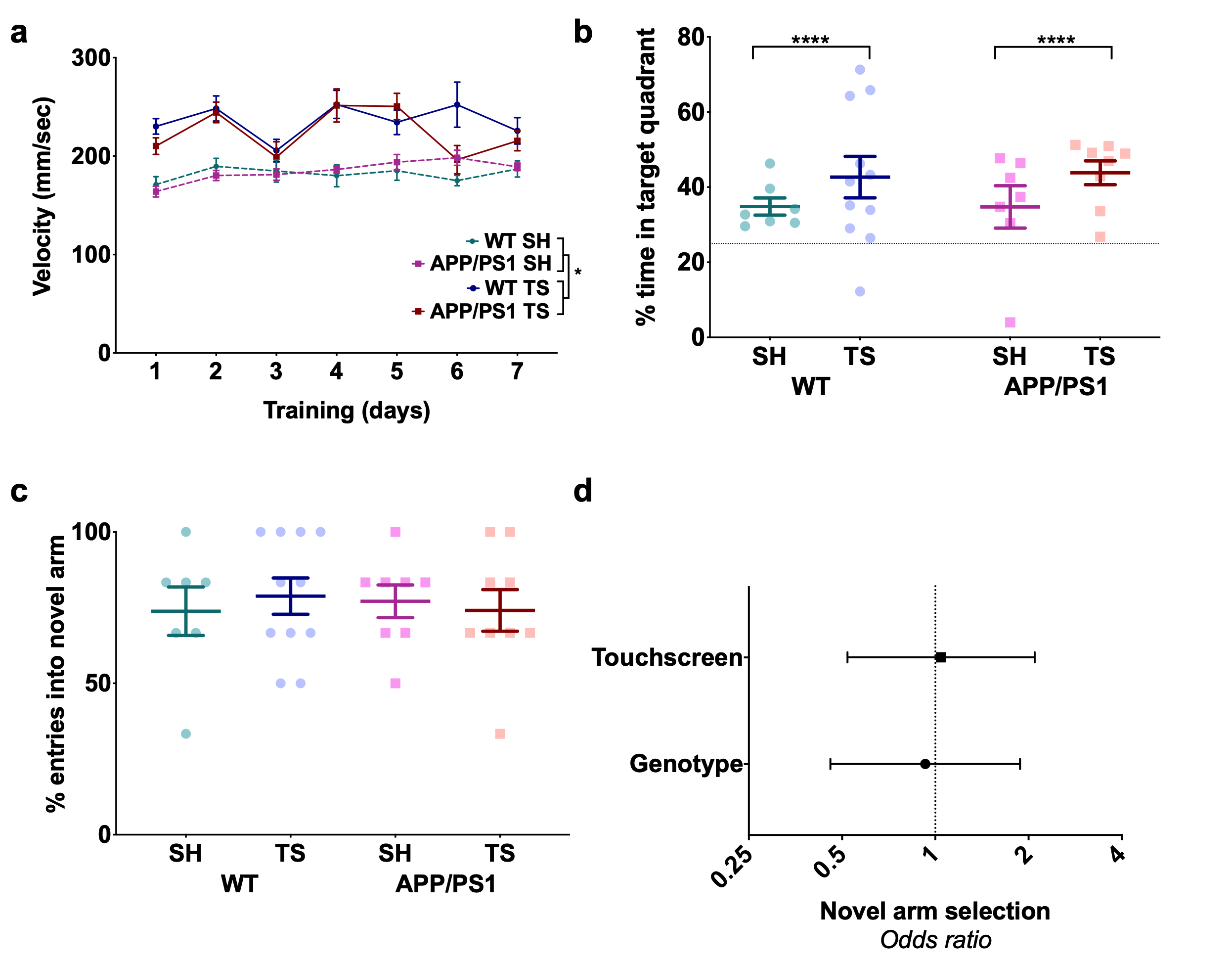

Supplement: Supplementary Figure 3 — Touchscreen-tested mice swim more quickly and show a stronger target quadrant preference than their standard housed counterparts in the MWM. (a) Both WT and APP/PS1 touchscreen-trained mice showed a higher swimming velocity in the MWM training across all days excepting day 3 and 6. (b) All groups showed a preference for the target quadrant during the MWM probe trial, with both WT and APP/PS1 touchscreen-trained groups showing a stronger preference than their standard-housed, behaviorally naïve counterparts. (c) All groups showed a strong preference for the novel arm during a discrete trial forced alternation task. (d) Neither genotype nor prior touchscreen exposure altered the odds of selecting the novel arm during discrete trial forced alternation. (a) Is presented as group mean ± SEM over 7 days of training. (b,c) Are presented as mean ± SEM with individual animals as dots. (d) Is the effect size ± 95% CI, showing the effect of genotype and prior touchscreen exposure on odds of novel arm selection. Dotted line in (b,c) represents chance performance (50%). *p < 0.05; ****p < 0.0001. [file Image_3.TIFF]

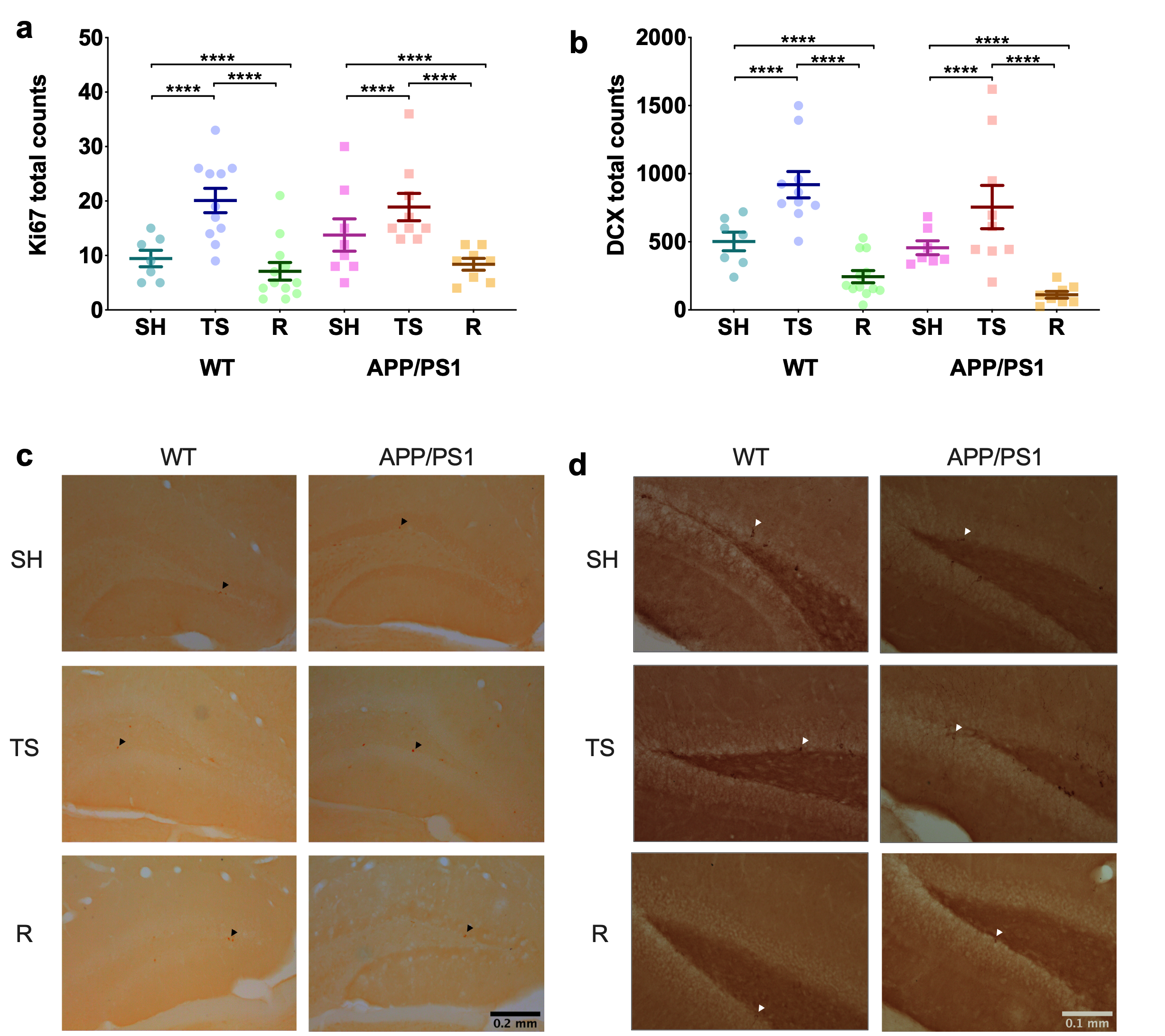

Supplement: Supplementary Figure 4 — High numbers of proliferating cells and immature neurons are not maintained in mice rested after completing touchscreen testing. (a) Total Ki67 counts are elevated following touchscreen training (TS) in both WT and APP/PS1 mice compared to standard housed (SH) animals, but this effect is lost when animals are rested (R) for 5 months following touchscreen training. (b) Total DCX counts are elevated following touchscreen training in both WT and APP/PS1 mice compared to SH animals, but this effect is lost when animals are rested for 5 months following touchscreen training. (c) Representative images of Ki67 DAB staining in WT and APP/PS1 mice that were SH, underwent TS training or were R following touchscreen training. (d) Representative images of DCX DAB staining in WT and APP/PS1 mice that were SH, underwent TS training or were R following touchscreen training. (a,b) Are presented as mean ± SEM with individual animals as dots. ****p < 0.0001. [file Image_4.TIFF]
