# Supplementary material for: A Preclinical Model of Computerized Cognitive Training: Touchscreen Cognitive Testing Enhances Cognition and Hippocampal Cellular Plasticity in Wildtype and Alzheimer’s Disease Mice
Source: Front Behav Neurosci. 2021 Dec 6;15:766745. doi: 10.3389/fnbeh.2021.766745 (PMC8685297; doi:10.3389/fnbeh.2021.766745)
Supplement: Supplementary file 5 [file Table_1.pdf]

Training Stage	Cohort	Age (months)	Dependent variable	Independent Variable	OR/IRR	p-value	95% Conf. Interval	
PAL	2	9.5-11.5	Correct selection	genotype	1.183	0.218	0.905	1.547
PAL	2	9.5-11.5	Correct selection	day	1.015	<0.0001	1.01	1.021
PAL	2	9.5-11.5	Correction trial	genotype	0.897	<0.0001	0.887	0.907
PAL	2	9.5-11.5	Correction trial	day	0.98	<0.0001	0.975	0.984
TUNL Stage 1	1	8-10	Correct selection	genotype	0.899	0.525	0.647	1.249
TUNL Stage 1	1	8-10	Correct selection	separation	0.977	0.758	0.845	1.131
TUNL Stage 1	1	8-10	Correct selection	day	1.022	0.068	0.998	1.047
TUNL Stage 1	2	13-14.5	Correct selection	genotype	1.052	0.742	0.777	1.426
TUNL Stage 1	2	13-14.5	Correct selection	separation	1.067	0.264	0.952	1.195
TUNL Stage 1	2	13-14.5	Correct selection	day	1.031	0.09	0.995	1.068
TUNL Stage 1	1	8-10	Correction trial	genotype	0.984	0.87	0.809	1.197
TUNL Stage 1	1	8-10	Correction trial	separation	1.467	<0.0001	1.316	1.636
TUNL Stage 1	1	8-10	Correction trial	day	1.013	0.067	0.999	1.027
TUNL Stage 1	2	13-14.5	Correction trial	genotype	1.018	0.888	0.79	1.313
TUNL Stage 1	2	13-14.5	Correction trial	separation	0.954	0.416	0.852	1.068
TUNL Stage 1	2	13-14.5	Correction trial	day	0.975	0.157	0.941	1.01
TUNL Stage 2	1	10-10.5	Correct selection	genotype	1.128	0.43	0.837	1.52
TUNL Stage 2	1	10-10.5	Correct selection	separation	1.077	0.35	0.922	1.259
TUNL Stage 2	1	10-10.5	Correct selection	day	0.978	0.069	0.955	1.002
TUNL Stage 2	2	14.5-15.5	Correct selection	genotype	1.02	0.85	0.831	1.251
TUNL Stage 2	2	14.5-15.5	Correct selection	separation	1.494	<0.0001	1.262	1.769
TUNL Stage 2	2	14.5-15.5	Correct selection	day	1.011	0.035	1.001	1.022
TUNL Stage 2	1	10-10.5	Correction trial	genotype	0.981	0.859	0.798	1.206
TUNL Stage 2	1	10-10.5	Correction trial	separation	0.751	<0.0001	0.648	0.869
TUNL Stage 2	1	10-10.5	Correction trial	day	0.98	0.02	0.963	0.997
TUNL Stage 2	2	14.5-15.5	Correction trial	genotype	1.01	0.882	0.888	1.149
TUNL Stage 2	2	14.5-15.5	Correction trial	separation	0.634	<0.0001	0.514	0.783
TUNL Stage 2	2	14.5-15.5	Correction trial	day	0.982	0.008	0.969	0.995
TUNL PS probe	1	10.5-11.5	Correct selection	genotype	0.897	0.463	0.671	1.199
TUNL PS probe	1	10.5-11.5	Correct selection	separation	1.465	0	1.298	1.654
TUNL PS probe	1	10.5-11.5	Correct selection	day	1.011	0.574	0.974	1.049
TUNL PS probe	2	15.5-16.5	Correct selection	genotype	0.989	0.914	0.816	1.199
TUNL PS probe	2	15.5-16.5	Correct selection	separation	1.311	0	1.145	1.5
TUNL PS probe	2	15.5-16.5	Correct selection	day	1.028	0.016	1.005	1.052
TUNL WM probe	1	10.5-11.5	Correct selection	genotype	0.99	0.898	0.857	1.145

TUNL WM probe	1	10.5-11.5	Correct selection	delay	0.859	0	0.838	0.881
TUNL WM probe	1	10.5-11.5	Correct selection	day	1.012	0.42	0.983	1.041
TUNL WM probe	2	15.5-16.5	Correct selection	genotype	1.187	0.124	0.954	1.476
TUNL WM probe	2	15.5-16.5	Correct selection	delay	0.854	0	0.837	0.872
TUNL WM probe	2	15.5-16.5	Correct selection	day	1.028	0	1.014	1.041

Supplementary Table 1. GLLAMM regression model statistics by task stage and cohort for PAL and TUNL.
